# {4,4′-Di­bromo-2,2′-[cyclo­hexane-1,2-diylbis(nitrilo­methanylyl­idene)]diphenolato-κ^4^ *O*,*N*,*N*′,*O*′}nickel(II)

**DOI:** 10.1107/S2414314621000845

**Published:** 2021-01-29

**Authors:** Kwang Ha

**Affiliations:** a Chonnam National University, School of Chemical Engineering, Research Institute of Catalysis, Gwangju, Republic of Korea; University of Otago, New Zealand

**Keywords:** crystal structure, nickel(II) complex, square-planar structure, Schiff base ligand

## Abstract

In the title compound, [Ni(C_20_H_18_Br_2_N_2_O_2_)], the Ni^II^ ion is four-coordinated in a slightly distorted square-planar coordination geometry defined by two N atoms and two O atoms of the tetra­dentate dianionic 4,4′-di­bromo-2,2′-[cyclo­hexane-1,2-diylbis(nitrilo­methanylyl­idene)]diphenolato ligand. Pairs of complex mol­ecules are assembled by inter­molecular C—H⋯O hydrogen bonds with *d*(C⋯O) = 3.247 (4) Å.

## Abstract 2

The central Ni^II^ ion has an N_2_O_2_ square-planar coordination sphere defined by two N atoms and two O atoms of the tetra­dentate dianionic 4,4′-di­bromo-2,2′-[cyclo­hexane-1,2-diylbis(nitrilo­methanylyl­idene)]diphenolato ligand.

## Graphical abstract

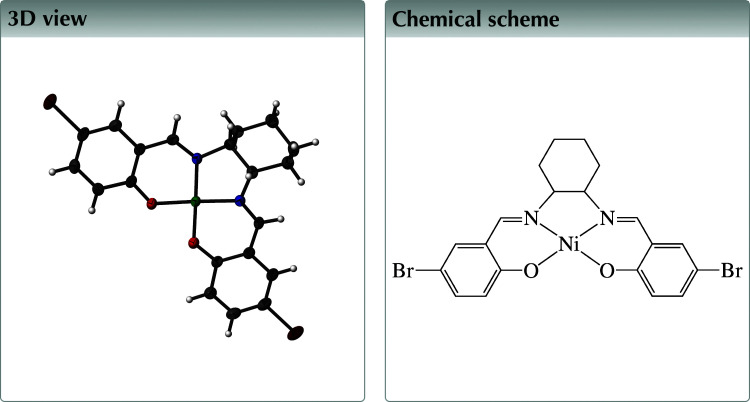

## Structure description

With reference to the title compound, [Ni(saldach)] (saldach = 4,4′-di­bromo- 2,2′-[cyclo­hexane-1,2-diylbis(nitrilo­methanylyl­idene)]diphenolato), the crystal structures of the tetra­dentate Schiff base (H_2_-saldach) ligand (Yi & Hu, 2009 ▸; Ha, 2012 ▸), and related saldach–metal complexes [Cu(saldach)] (Tohidiyan *et al.*, 2017 ▸) and [Zn(saldach)(pyridine)] (Szłyk *et al.*, 2005 ▸) have been determined previously.

**Figure 1:**
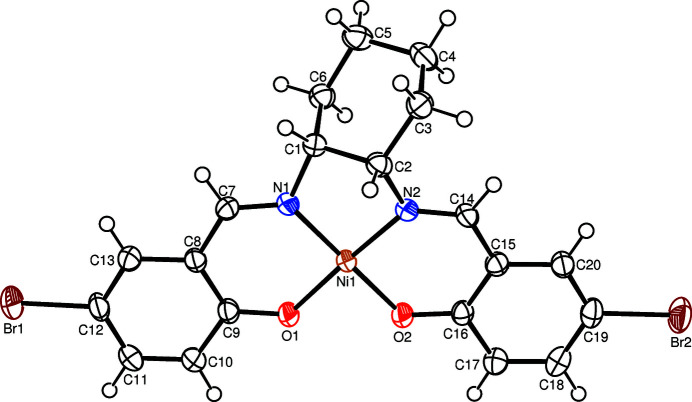
The mol­ecular structure of the title compound showing the atom labelling and displacement ellipsoids drawn at the 50% probability level for non-H atoms.

**Figure 2:**
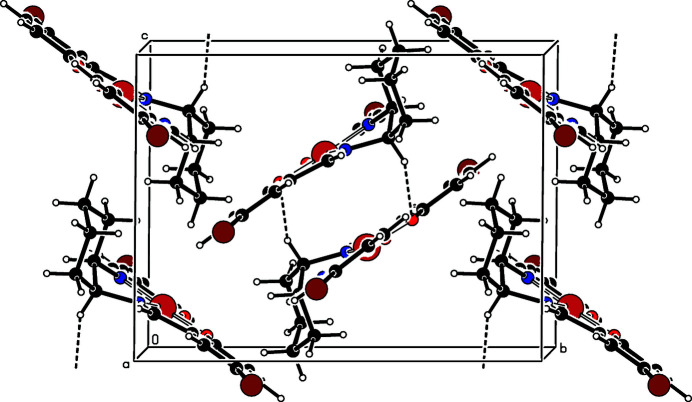
The packing in the crystal structure of the title compound, viewed approximately along the *a* axis. Hydrogen-bonding inter­actions are drawn as dashed lines.

In the title complex, the central Ni^II^ cation is four-coordinated in a slightly distorted square-planar coordination geometry defined by the N1, N2, O1 and O2 atoms of the tetra­dentate dianionic saldach ligand (Fig. 1 ▸). The tight N—Ni—N and N—Ni—O chelating angles of <N1—Ni1—N2 = 86.13 (10)°, <N1—Ni1—O1 = 94.64 (10)° and <N2—Ni1—O2 = 95.02 (10)° form the square-plane. The Ni—N and Ni—O bonds are almost equal [1.844 (2)–1.858 (2) Å] and the nearly planar benzene rings of the saldach ligand are slightly twisted with a dihedral angle of 2.9 (2)° between them. The dihedral angles between the least-squares plane [maximum deviation = 0.066 (1) Å] of the Ni square-plane (Ni1/O1/O2/N1/N2) and the benzene rings are 7.2 (2) and 4.4 (2)°, respectively. In the crystal structure (Fig. 2 ▸), pairs of complex mol­ecules are assembled by inter­molecular C—H⋯O hydrogen bonds (Table 1 ▸). In addition, the complex displays several inter­molecular π–π inter­actions between adjacent benzene rings. For *Cg*1 (the centroid of ring C8–C13) and *Cg*2^i^ [the centroid of ring C15—C20; symmetry code: (i) −*x* + 1, −*y* + 1, −*z* + 1], the centroid–centroid distance is 4.081 (2) Å and the dihedral angle between the ring planes is 2.9 (1)°.

## Synthesis and crystallization

To a solution of Ni(acac)_2_ (acac = pentane-2,4-dionate; 0.1231 g, 0.479 mmol) in acetone (30 ml) was added 4,4′-di­bromo-2,2′-[cyclo­hexane-1,2-diylbis(nitrilo­methanylyl­idene)]diphenol (0.2320 g, 0.483 mmol; Ha, 2012 ▸) and stirred for 1 h at room temperature. After addition of ether (30 ml), the formed precipitate was separated by filtration, washed with ether, and dried at 323 K, to give a brown powder (0.2464 g). Brown crystals suitable for X-ray analysis were obtained by slow evaporation from a dimethyl sulfoxide (DMSO) solution at 363 K.

## Refinement

Crystal data, data collection and structure refinement details are summarized in Table 2 ▸. The highest peak (0.77 e Å^−3^) and the deepest hole (−0.51 e Å^−3^) in the difference Fourier map are located 0.86 and 0.70 Å, respectively, from the atoms Br1 and Br2.

## Supplementary Material

Crystal structure: contains datablock(s) I. DOI: 10.1107/S2414314621000845/sj4220sup1.cif

Structure factors: contains datablock(s) I. DOI: 10.1107/S2414314621000845/sj4220Isup2.hkl

CCDC reference: 2058387

Additional supporting information:  crystallographic information; 3D view; checkCIF report

## Figures and Tables

**Table 1:** Hydrogen-bond geometry (Å, °)

*D*—H⋯*A*	*D*—H	H⋯*A*	*D*⋯*A*	*D*—H⋯*A*
C2—H2⋯O2^i^	0.99	2.36	3.247 (4)	149

Symmetry code: (i) 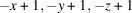 .

**Table 2:** Experimental details

Crystal data	
Chemical formula	[Ni(C_20_H_18_Br_2_N_2_O_2_)]
*M*_r_	536.89
Crystal system, space group	Monoclinic, *P*2_1_/*c*
Temperature (K)	223
*a*, *b*, *c* (Å)	13.5179 (4), 13.6343 (5), 10.4966 (4)
β (°)	104.510 (1)
*V* (Å^3^)	1872.89 (11)
*Z*	4
Radiation type	Mo *K*α
μ (mm^−1^)	5.32
Crystal size (mm)	0.14 × 0.10 × 0.07

Data collection	
Diffractometer	PHOTON 100 CMOS detector
Absorption correction	Multi-scan (*SADABS*; Bruker, 2016 ▸)
*T*_min_, *T*_max_	0.618, 0.745
No. of measured, independent and observed [*I* > 2σ(*I*)] reflections	50588, 3710, 3107
*R*_int_	0.059
(sin θ/λ)_max_ (Å^−1^)	0.619

Refinement	
*R*[*F* ^2^ > 2σ(*F* ^2^)], *wR*(*F* ^2^), *S*	0.032, 0.072, 1.10
No. of reflections	3710
No. of parameters	244
H-atom treatment	H-atom parameters constrained
Δρ_max_, Δρ_min_ (e Å^−3^)	0.77, −0.51

Computer programs: *APEX2* and *SAINT* (Bruker, 2016 ▸), *SHELXT2014/7* (Sheldrick, 2015*a* ▸), *SHELXL2014/7* (Sheldrick, 2015*b* ▸) and *ORTEP-3 for Windows* (Farrugia, 2012 ▸).

## full crystallographic data

### Crystal data

**Table:**

[Ni(C_20_H_18_Br_2_N_2_O_2_)]	*F*(000) = 1064
*M**_r_* = 536.89	*D*_x_ = 1.904 Mg m^−^^3^
Monoclinic, *P*2_1_/*c*	Mo *K*α radiation, λ = 0.71073 Å
*a* = 13.5179 (4) Å	Cell parameters from 9977 reflections
*b* = 13.6343 (5) Å	θ = 2.5–26.0°
*c* = 10.4966 (4) Å	µ = 5.32 mm^−^^1^
β = 104.510 (1)°	*T* = 223 K
*V* = 1872.89 (11) Å^3^	Block, brown
*Z* = 4	0.14 × 0.10 × 0.07 mm

### Data collection

**Table:**

PHOTON 100 CMOS detector diffractometer	3107 reflections with *I* > 2σ(*I*)
Radiation source: sealed tube	*R*_int_ = 0.059
φ and ω scans	θ_max_ = 26.1°, θ_min_ = 2.2°
Absorption correction: multi-scan (SADABS; Bruker, 2016)	*h* = −16→16
*T*_min_ = 0.618, *T*_max_ = 0.745	*k* = −16→16
50588 measured reflections	*l* = −12→12
3710 independent reflections	

### Refinement

**Table:**

Refinement on *F*^2^	Primary atom site location: structure-invariant direct methods
Least-squares matrix: full	Secondary atom site location: difference Fourier map
*R*[*F*^2^ > 2σ(*F*^2^)] = 0.032	Hydrogen site location: inferred from neighbouring sites
*wR*(*F*^2^) = 0.072	H-atom parameters constrained
*S* = 1.10	*w* = 1/[σ^2^(*F*_o_^2^) + (0.0258*P*)^2^ + 2.4859*P*] where *P* = (*F*_o_^2^ + 2*F*_c_^2^)/3
3710 reflections	(Δ/σ)_max_ = 0.002
244 parameters	Δρ_max_ = 0.77 e Å^−^^3^
0 restraints	Δρ_min_ = −0.51 e Å^−^^3^

### Special details

**Table:**

Geometry. All esds (except the esd in the dihedral angle between two l.s. planes) are estimated using the full covariance matrix. The cell esds are taken into account individually in the estimation of esds in distances, angles and torsion angles; correlations between esds in cell parameters are only used when they are defined by crystal symmetry. An approximate (isotropic) treatment of cell esds is used for estimating esds involving l.s. planes.
Refinement. Hydrogen atoms on C atoms were positioned geometrically and allowed to ride on their respective parent atoms: C—H = 0.94, 0.98 or 0.99 Å and *U*_iso_(H) = 1.2*U*_eq_(C).

### Fractional atomic coordinates and isotropic or equivalent isotropic displacement parameters (Å^2^)

**Table:**

	*x*	*y*	*z*	*U*_iso_*/*U*_eq_	
Br1	1.00079 (3)	0.44652 (3)	0.23580 (4)	0.05730 (14)	
Br2	0.08652 (3)	0.78593 (3)	0.58203 (4)	0.05165 (13)	
Ni1	0.49753 (3)	0.55203 (3)	0.34846 (4)	0.02442 (10)	
O1	0.62893 (15)	0.60079 (16)	0.3841 (2)	0.0304 (5)	
O2	0.47131 (15)	0.66161 (16)	0.4383 (2)	0.0301 (5)	
N1	0.52417 (17)	0.44688 (18)	0.2516 (2)	0.0252 (5)	
N2	0.36814 (17)	0.49806 (19)	0.3268 (2)	0.0267 (5)	
C1	0.4343 (2)	0.3849 (2)	0.1927 (3)	0.0269 (6)	
H1	0.4565	0.3161	0.1883	0.032*	
C2	0.3649 (2)	0.3921 (2)	0.2871 (3)	0.0284 (7)	
H2	0.3975	0.3537	0.3667	0.034*	
C3	0.2600 (2)	0.3463 (3)	0.2275 (3)	0.0339 (7)	
H3A	0.2675	0.2748	0.2255	0.041*	
H3B	0.2146	0.3613	0.2844	0.041*	
C4	0.2111 (2)	0.3822 (3)	0.0896 (3)	0.0374 (8)	
H4A	0.1956	0.4523	0.0921	0.045*	
H4B	0.1468	0.3471	0.0545	0.045*	
C5	0.2827 (2)	0.3653 (3)	0.0006 (3)	0.0361 (8)	
H5A	0.2505	0.3886	−0.0884	0.043*	
H5B	0.2966	0.2950	−0.0041	0.043*	
C6	0.3832 (2)	0.4208 (2)	0.0554 (3)	0.0308 (7)	
H6A	0.4290	0.4105	−0.0025	0.037*	
H6B	0.3693	0.4912	0.0579	0.037*	
C7	0.6106 (2)	0.4235 (2)	0.2282 (3)	0.0265 (6)	
H7	0.6122	0.3676	0.1762	0.032*	
C8	0.7038 (2)	0.4763 (2)	0.2755 (3)	0.0255 (6)	
C9	0.7083 (2)	0.5611 (2)	0.3546 (3)	0.0271 (7)	
C10	0.8051 (2)	0.6037 (2)	0.4047 (3)	0.0325 (7)	
H10	0.8112	0.6575	0.4622	0.039*	
C11	0.8910 (2)	0.5690 (3)	0.3719 (3)	0.0370 (8)	
H11	0.9545	0.5994	0.4058	0.044*	
C12	0.8835 (2)	0.4889 (3)	0.2886 (3)	0.0336 (7)	
C13	0.7927 (2)	0.4414 (2)	0.2423 (3)	0.0304 (7)	
H13	0.7892	0.3857	0.1887	0.036*	
C14	0.2916 (2)	0.5380 (2)	0.3591 (3)	0.0290 (7)	
H14	0.2294	0.5035	0.3390	0.035*	
C15	0.2940 (2)	0.6313 (2)	0.4236 (3)	0.0268 (6)	
C16	0.3855 (2)	0.6850 (2)	0.4669 (3)	0.0255 (6)	
C17	0.3841 (2)	0.7673 (2)	0.5479 (3)	0.0310 (7)	
H17	0.4446	0.8031	0.5803	0.037*	
C18	0.2964 (3)	0.7962 (2)	0.5805 (3)	0.0347 (7)	
H18	0.2974	0.8511	0.6350	0.042*	
C19	0.2063 (2)	0.7446 (2)	0.5329 (3)	0.0332 (7)	
C20	0.2041 (2)	0.6637 (2)	0.4562 (3)	0.0316 (7)	
H20	0.1426	0.6293	0.4248	0.038*	

### Atomic displacement parameters (Å^2^)

**Table:**

	*U*^11^	*U*^22^	*U*^33^	*U*^12^	*U*^13^	*U*^23^
Br1	0.02496 (17)	0.0832 (3)	0.0682 (3)	0.00022 (18)	0.02006 (17)	−0.0224 (2)
Br2	0.0399 (2)	0.0644 (3)	0.0559 (2)	0.01865 (18)	0.02165 (17)	−0.0045 (2)
Ni1	0.01809 (18)	0.0301 (2)	0.0257 (2)	−0.00166 (15)	0.00659 (15)	−0.00440 (17)
O1	0.0212 (10)	0.0346 (12)	0.0371 (13)	−0.0038 (9)	0.0107 (9)	−0.0096 (10)
O2	0.0239 (10)	0.0335 (12)	0.0350 (12)	−0.0034 (9)	0.0112 (9)	−0.0075 (10)
N1	0.0207 (12)	0.0302 (13)	0.0250 (13)	−0.0024 (10)	0.0061 (10)	−0.0024 (11)
N2	0.0220 (12)	0.0334 (15)	0.0245 (13)	−0.0024 (11)	0.0055 (10)	−0.0044 (11)
C1	0.0238 (14)	0.0274 (16)	0.0292 (16)	−0.0021 (12)	0.0062 (12)	−0.0055 (13)
C2	0.0260 (15)	0.0305 (17)	0.0283 (17)	−0.0044 (13)	0.0059 (12)	−0.0003 (13)
C3	0.0322 (17)	0.0345 (18)	0.0372 (19)	−0.0062 (14)	0.0129 (14)	−0.0041 (15)
C4	0.0233 (15)	0.041 (2)	0.044 (2)	−0.0023 (14)	0.0019 (14)	−0.0008 (16)
C5	0.0352 (17)	0.043 (2)	0.0266 (17)	−0.0046 (15)	0.0018 (14)	0.0005 (15)
C6	0.0305 (16)	0.0333 (18)	0.0289 (17)	−0.0022 (13)	0.0077 (13)	−0.0010 (14)
C7	0.0265 (15)	0.0311 (17)	0.0220 (15)	0.0022 (12)	0.0067 (12)	−0.0017 (12)
C8	0.0216 (14)	0.0326 (17)	0.0226 (15)	0.0024 (12)	0.0061 (12)	0.0028 (13)
C9	0.0217 (14)	0.0340 (17)	0.0269 (16)	0.0018 (13)	0.0084 (12)	0.0019 (13)
C10	0.0256 (15)	0.0359 (18)	0.0350 (18)	−0.0018 (13)	0.0059 (13)	−0.0063 (15)
C11	0.0211 (15)	0.048 (2)	0.041 (2)	−0.0033 (14)	0.0053 (14)	−0.0051 (16)
C12	0.0202 (14)	0.046 (2)	0.0361 (19)	0.0059 (14)	0.0092 (13)	0.0017 (15)
C13	0.0273 (15)	0.0382 (18)	0.0263 (16)	0.0037 (14)	0.0077 (13)	−0.0019 (14)
C14	0.0202 (14)	0.0404 (19)	0.0269 (16)	−0.0041 (13)	0.0066 (12)	−0.0025 (14)
C15	0.0262 (15)	0.0322 (17)	0.0227 (15)	0.0013 (13)	0.0073 (12)	0.0025 (13)
C16	0.0257 (14)	0.0286 (16)	0.0237 (15)	0.0012 (12)	0.0092 (12)	0.0038 (13)
C17	0.0321 (16)	0.0277 (17)	0.0349 (18)	0.0002 (13)	0.0117 (14)	0.0003 (14)
C18	0.0433 (19)	0.0314 (18)	0.0342 (18)	0.0060 (15)	0.0186 (15)	0.0016 (14)
C19	0.0322 (16)	0.0380 (19)	0.0322 (18)	0.0116 (14)	0.0136 (14)	0.0070 (15)
C20	0.0251 (15)	0.0403 (19)	0.0299 (17)	0.0033 (14)	0.0081 (13)	0.0011 (14)

### Geometric parameters (Å, º)

**Table:**

Br1—C12	1.896 (3)	C5—H5B	0.9800
Br2—C19	1.903 (3)	C6—H6A	0.9800
Ni1—N1	1.844 (2)	C6—H6B	0.9800
Ni1—O1	1.8449 (19)	C7—C8	1.429 (4)
Ni1—O2	1.848 (2)	C7—H7	0.9400
Ni1—N2	1.858 (2)	C8—C13	1.414 (4)
O1—C9	1.307 (3)	C8—C9	1.415 (4)
O2—C16	1.309 (3)	C9—C10	1.407 (4)
N1—C7	1.293 (4)	C10—C11	1.375 (4)
N1—C1	1.482 (4)	C10—H10	0.9400
N2—C14	1.288 (4)	C11—C12	1.386 (5)
N2—C2	1.501 (4)	C11—H11	0.9400
C1—C6	1.515 (4)	C12—C13	1.365 (4)
C1—C2	1.528 (4)	C13—H13	0.9400
C1—H1	0.9900	C14—C15	1.437 (4)
C2—C3	1.532 (4)	C14—H14	0.9400
C2—H2	0.9900	C15—C16	1.411 (4)
C3—C4	1.514 (5)	C15—C20	1.413 (4)
C3—H3A	0.9800	C16—C17	1.412 (4)
C3—H3B	0.9800	C17—C18	1.371 (4)
C4—C5	1.522 (5)	C17—H17	0.9400
C4—H4A	0.9800	C18—C19	1.386 (5)
C4—H4B	0.9800	C18—H18	0.9400
C5—C6	1.534 (4)	C19—C20	1.361 (5)
C5—H5A	0.9800	C20—H20	0.9400
			
N1—Ni1—O1	94.64 (10)	C5—C6—H6A	109.6
N1—Ni1—O2	177.02 (10)	C1—C6—H6B	109.6
O1—Ni1—O2	84.45 (9)	C5—C6—H6B	109.6
N1—Ni1—N2	86.13 (10)	H6A—C6—H6B	108.1
O1—Ni1—N2	175.08 (11)	N1—C7—C8	124.7 (3)
O2—Ni1—N2	95.02 (10)	N1—C7—H7	117.6
C9—O1—Ni1	127.51 (19)	C8—C7—H7	117.6
C16—O2—Ni1	127.46 (19)	C13—C8—C9	120.3 (3)
C7—N1—C1	117.7 (2)	C13—C8—C7	118.3 (3)
C7—N1—Ni1	127.4 (2)	C9—C8—C7	121.4 (3)
C1—N1—Ni1	114.85 (18)	O1—C9—C10	118.8 (3)
C14—N2—C2	120.7 (3)	O1—C9—C8	124.1 (3)
C14—N2—Ni1	126.5 (2)	C10—C9—C8	117.1 (3)
C2—N2—Ni1	112.07 (18)	C11—C10—C9	121.9 (3)
N1—C1—C6	110.1 (2)	C11—C10—H10	119.1
N1—C1—C2	105.4 (2)	C9—C10—H10	119.1
C6—C1—C2	112.9 (2)	C10—C11—C12	119.7 (3)
N1—C1—H1	109.5	C10—C11—H11	120.1
C6—C1—H1	109.5	C12—C11—H11	120.1
C2—C1—H1	109.5	C13—C12—C11	121.0 (3)
N2—C2—C1	105.2 (2)	C13—C12—Br1	119.7 (3)
N2—C2—C3	117.7 (3)	C11—C12—Br1	119.3 (2)
C1—C2—C3	111.4 (2)	C12—C13—C8	119.7 (3)
N2—C2—H2	107.4	C12—C13—H13	120.1
C1—C2—H2	107.4	C8—C13—H13	120.1
C3—C2—H2	107.4	N2—C14—C15	125.0 (3)
C4—C3—C2	113.4 (3)	N2—C14—H14	117.5
C4—C3—H3A	108.9	C15—C14—H14	117.5
C2—C3—H3A	108.9	C16—C15—C20	119.8 (3)
C4—C3—H3B	108.9	C16—C15—C14	121.7 (3)
C2—C3—H3B	108.9	C20—C15—C14	118.2 (3)
H3A—C3—H3B	107.7	O2—C16—C15	123.8 (3)
C3—C4—C5	110.2 (3)	O2—C16—C17	118.6 (3)
C3—C4—H4A	109.6	C15—C16—C17	117.6 (3)
C5—C4—H4A	109.6	C18—C17—C16	121.4 (3)
C3—C4—H4B	109.6	C18—C17—H17	119.3
C5—C4—H4B	109.6	C16—C17—H17	119.3
H4A—C4—H4B	108.1	C17—C18—C19	120.1 (3)
C4—C5—C6	109.6 (3)	C17—C18—H18	120.0
C4—C5—H5A	109.8	C19—C18—H18	120.0
C6—C5—H5A	109.8	C20—C19—C18	120.7 (3)
C4—C5—H5B	109.8	C20—C19—Br2	120.4 (3)
C6—C5—H5B	109.8	C18—C19—Br2	118.9 (2)
H5A—C5—H5B	108.2	C19—C20—C15	120.3 (3)
C1—C6—C5	110.3 (3)	C19—C20—H20	119.9
C1—C6—H6A	109.6	C15—C20—H20	119.9
			
N1—Ni1—O1—C9	−5.3 (3)	Ni1—O1—C9—C10	−172.2 (2)
O2—Ni1—O1—C9	177.5 (3)	Ni1—O1—C9—C8	7.2 (4)
O1—Ni1—O2—C16	−176.7 (3)	C13—C8—C9—O1	176.5 (3)
N2—Ni1—O2—C16	−1.6 (3)	C7—C8—C9—O1	−4.1 (5)
O1—Ni1—N1—C7	1.5 (3)	C13—C8—C9—C10	−4.1 (4)
N2—Ni1—N1—C7	−173.6 (3)	C7—C8—C9—C10	175.3 (3)
O1—Ni1—N1—C1	−176.9 (2)	O1—C9—C10—C11	−176.4 (3)
N2—Ni1—N1—C1	8.0 (2)	C8—C9—C10—C11	4.1 (5)
N1—Ni1—N2—C14	−172.0 (3)	C9—C10—C11—C12	−0.8 (5)
O2—Ni1—N2—C14	5.3 (3)	C10—C11—C12—C13	−2.6 (5)
N1—Ni1—N2—C2	17.9 (2)	C10—C11—C12—Br1	176.3 (3)
O2—Ni1—N2—C2	−164.82 (19)	C11—C12—C13—C8	2.5 (5)
C7—N1—C1—C6	−87.0 (3)	Br1—C12—C13—C8	−176.4 (2)
Ni1—N1—C1—C6	91.6 (2)	C9—C8—C13—C12	1.0 (5)
C7—N1—C1—C2	151.0 (3)	C7—C8—C13—C12	−178.5 (3)
Ni1—N1—C1—C2	−30.4 (3)	C2—N2—C14—C15	166.6 (3)
C14—N2—C2—C1	151.3 (3)	Ni1—N2—C14—C15	−2.7 (5)
Ni1—N2—C2—C1	−38.0 (3)	N2—C14—C15—C16	−4.9 (5)
C14—N2—C2—C3	26.6 (4)	N2—C14—C15—C20	−178.1 (3)
Ni1—N2—C2—C3	−162.6 (2)	Ni1—O2—C16—C15	−4.8 (4)
N1—C1—C2—N2	41.5 (3)	Ni1—O2—C16—C17	173.8 (2)
C6—C1—C2—N2	−78.7 (3)	C20—C15—C16—O2	−178.2 (3)
N1—C1—C2—C3	170.1 (2)	C14—C15—C16—O2	8.8 (5)
C6—C1—C2—C3	49.8 (4)	C20—C15—C16—C17	3.2 (4)
N2—C2—C3—C4	71.7 (4)	C14—C15—C16—C17	−169.8 (3)
C1—C2—C3—C4	−49.8 (4)	O2—C16—C17—C18	179.4 (3)
C2—C3—C4—C5	55.3 (4)	C15—C16—C17—C18	−2.0 (5)
C3—C4—C5—C6	−59.6 (4)	C16—C17—C18—C19	−0.4 (5)
N1—C1—C6—C5	−172.9 (2)	C17—C18—C19—C20	1.5 (5)
C2—C1—C6—C5	−55.5 (3)	C17—C18—C19—Br2	179.2 (2)
C4—C5—C6—C1	59.9 (3)	C18—C19—C20—C15	−0.2 (5)
C1—N1—C7—C8	178.9 (3)	Br2—C19—C20—C15	−177.8 (2)
Ni1—N1—C7—C8	0.5 (5)	C16—C15—C20—C19	−2.3 (5)
N1—C7—C8—C13	179.6 (3)	C14—C15—C20—C19	171.0 (3)
N1—C7—C8—C9	0.2 (5)		

### Hydrogen-bond geometry (Å, º)

**Table:**

*D*—H···*A*	*D*—H	H···*A*	*D*···*A*	*D*—H···*A*
C2—H2···O2^i^	0.99	2.36	3.247 (4)	149

Symmetry code: (i) −*x*+1, −*y*+1, −*z*+1.

## References

[bb1] Bruker (2016). *APEX2*, *SAINT* and *SADABS*. Bruker AXS Inc., Madison, Wisconsin, USA.

[bb2] Farrugia, L. J. (2012). *J. Appl. Cryst.* **45**, 849–854.

[bb3] Ha, K. (2012). *Acta Cryst.* E**68**, o1449. 10.1107/S1600536812016376 PMC3344566 22590328

[bb4] Sheldrick, G. M. (2015*a*). *Acta Cryst.* A**71**, 3–8.

[bb5] Sheldrick, G. M. (2015*b*). *Acta Cryst.* C**71**, 3–8.

[bb6] Szłyk, E., Wojtczak, A., Surdykowski, A. & Goździkiewicz, M. (2005). *Inorg. Chim. Acta*, **358**, 467–475.

[bb7] Tohidiyan, Z., Sheikhshoaie, I., Khaleghi, M. & Mague, J. T. (2017). *J. Mol. Struct.* **1134**, 706–714.

[bb8] Yi, J. & Hu, S. (2009). *Acta Cryst.* E**65**, o2643. 10.1107/S1600536809039671 PMC2971247 21578257

